# scATAC-Seq reveals heterogeneity associated with spermatogonial differentiation in cultured male germline stem cells

**DOI:** 10.1038/s41598-022-25729-7

**Published:** 2022-12-12

**Authors:** Hoi Ching Suen, Alfred Chun Shui Luk, Jinyue Liao

**Affiliations:** 1 Developmental and Regenerative Biology Program, School of Biomedical Sciences, Faculty of Medicine, The Chinese University of Hong Kong, Shatin, Hong Kong SAR China; 2 Department of Chemical Pathology, Faculty of Medicine, The Chinese University of Hong Kong, Shatin, Hong Kong SAR China

**Keywords:** Epigenomics, Stem-cell differentiation

## Abstract

Spermatogonial stem cells are the most primitive spermatogonia in testis, which can self-renew to maintain the stem cell pool or differentiate to give rise to germ cells including haploid spermatids. All-trans-retinoic acid (RA), a bioactive metabolite of vitamin A, plays a fundamental role in initiating spermatogonial differentiation. In this study, single-cell ATAC-seq (scATAC-seq) was used to obtain genome-wide chromatin maps of cultured germline stem cells (GSCs) that were in control and RA-induced differentiation states. We showed that different subsets of GSCs can be distinguished based on chromatin accessibility of self-renewal and differentiation signature genes. Importantly, both progenitors and a subset of stem cells are able to respond to RA and give rise to differentiating cell subsets with distinct chromatin accessibility profiles. In this study, we identified regulatory regions that undergo chromatin remodeling and are associated with the retinoic signaling pathway. Moreover, we reconstructed the differentiation trajectory and identified novel transcription factor candidates enriched in different spermatogonia subsets. Collectively, our work provides a valuable resource for understanding the heterogeneity associated with differentiation and RA response in GSCs.

## Introduction

Spermatogenesis is a complicated and precisely regulated process in which mature spermatozoa are produced inside the seminiferous tubules. Haploid spermatozoa are derived from undifferentiated spermatogonia. The undifferentiated spermatogonia consist of A single (A_s_), A paired (A_pr_) and A aligned (A_al_) spermatogonia, including spermatogonial stem cells (SSCs) and progenitor cells. SSCs (A_s_) are the most primitive stem cell population, and can undergo active self-renewal or give rise to differentiation-primed progenitor cells by mitotic cell division (A_pr_ and A_al_)^1^. To initiate differentiation, A_al_ spermatogonia transit into A_1_ spermatogonia. Following a few more rounds of divisions, A_2_, A_3_, and A_4_ spermatogonia form, respectively. After that, A_4_ spermatogonia mature into intermediate and type B spermatogonia, which will divide into primary spermatocytes^1^.

In the undifferentiated spermatogonia population, spermatogonia markers can display a gradient of expression level and define different cellular states. Currently, the SSC population cannot be defined by a single marker but a panel of SSC self-renewal genes instead, such as *Gfra1*^2^, *Id4*^3^, *Egr2*^4^, *Lhx1*^4^, *Lhx2*^5^ and *Etv5*^6^. When it starts to transition to progenitor cells, genes such as *Upp1*, *Sox3*, *Ngn3*, *Nanos3*, *Ddit4* and *Piwil4* will be upregulated, and the self-renewal genes will be downregulated^5^. The gene expression of spermatogonia is therefore highly heterogeneous. During spermatogonial differentiation, genes associated with differentiation, such as *Kit* and *Stra8*, will be upregulated^7^.

All-trans-retinoic acid (RA) is a bioactive metabolite of vitamin A. Previous studies have found that RA signaling plays a fundamental role in initiating spermatogonial differentiation. Spermatogonia cannot reach the A1 stage from Aal stage in mice when RA signaling is blocked due to vitamin A-deficient (VAD) diet^8^. According to the "revised A_single_ model," spermatogonia's tendency to respond to the RA signal is proportional to chain length, and A_pr_ can also respond to RA^9,10^. The action of RA on target gene expression depends on two families of nuclear hormone receptors, the retinoic acid receptors (RARs) and the retinoid X receptors (RXRs). RARs usually function with RXRs to form heterodimers, which can bind to retinoic acid response elements (RAREs) of the genome to regulate RA-target gene expression^11^. In spermatogonia, RARG is the major functional RAR isotype and it can form heterodimer with RXRA^12^.

Recent single cell RNA-seq analysis has deepened our understanding of the cellular heterogeneity and transcriptome dynamics associated with spermatogonial differentiation. However, the dynamic remodeling of chromatin landscapes and differential transcription factors (TFs) motif usage during RA-induced spermatogonial differentiation have not been addressed. To initiate or regulate gene expression, TFs need to bind to their DNA binding sites at the promoter and enhancer DNA of the target gene first. The TF binding sequence is usually represented as binding site motifs, which indicate the preferentially bound sequences^13^.The chromatin in TF bound regions and gene loci of genes with active transcription is more open and accessible^14^. These regions demonstrate decreased nucleosomal density and are sometimes nucleosome-free^15^. ATAC-seq combined with TF binding motifs has proven increasingly effective for discovering dynamic changes in chromatin landscapes and predicting critical regulatory events that cause chromatin remodeling. While Maezawa et al. have provided much information on the changes in chromatin landscapes during spermatogenesis using bulk ATAC-seq, the THY1+ undifferentiated spermatogonia sample consisted of a mix of spermatogonial stem and progenitor cells^16^. Thus, the main aim of this pilot study was to assess the utility of single-cell ATAC-seq for uncovering cis-regulatory elements and TF regulators in spermatogonial differentiation, especially the transition from stem cells to progenitor cells, and then to differentiating spermatogonia.

In this study, we administered RA to GSCs culture and profiled the chromatin accessibility landscape by single-cell ATAC-seq (scATAC-seq). Our study shows that stem cell-like and progenitor-like cells display distinct chromatin accessibility profiles, with stem cell-like cells displaying more profound chromatin accessibility changes induced by RA. We also explored the regulatory regions in spermatogonial subpopulations after RA stimulation. We further reconstructed the pseudotime differentiation trajectory and revealed dynamics of TF activity associated with the transition. Collectively, our study provides novel insights on the epigenetic regulations of spermatogonial differentiation induced by RA.

## Results

### Differentiation of GSC in vitro

**Figure 1:**
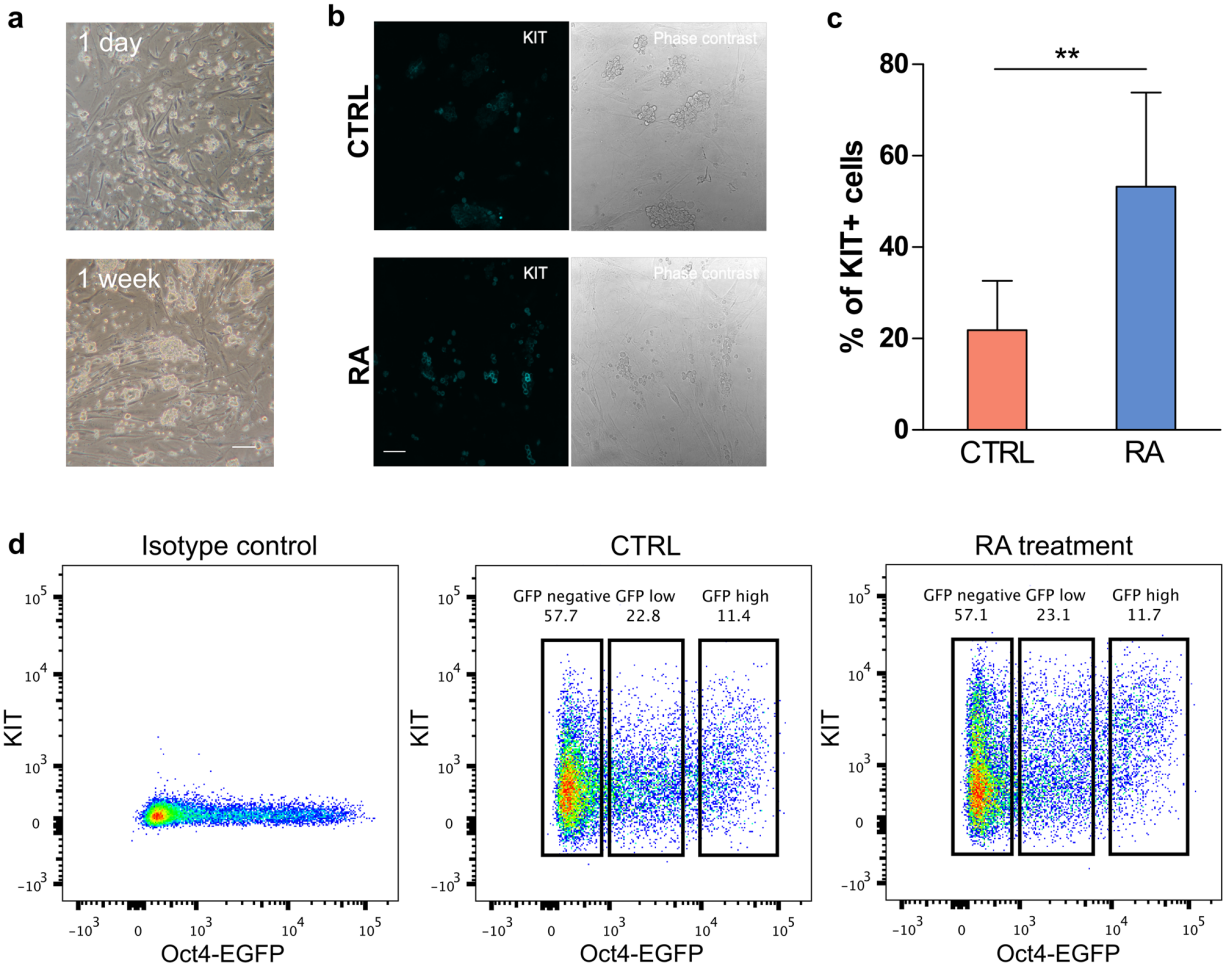
Retinoic acid (RA) induces differentiation in germline stem cells (GSCs) in vitro. (**a**) Bright field images of stabilized germline stem cell (GSC) culture on the MEF feeder layer showing 1 day after subculture (top) and 1 week after subculture (bottom). Scale bars 100 μm. (**b**) Representative phase contrast and immunofluorescence images of control (upper panel) and RA-treated (lower panel) GSC culture stained for KIT. Scale bars 50 μm. (**c**) Quantification of KIT + cells in RA-treated and control GSCs by flow cytometry analysis (P < 0.01, n = 6). Error bars indicate SD. Significance was determined by an unpaired two-tailed t-test. (**d**) Flow cytometry analysis of Oct4-EGFP and KIT levels in GSCs treated with 0.5 μM RA for 24 h, DMSO control and unstained isotype control. Cells were stained with mouse anti-KIT-APC antibody.

To facilitate the investigation of the differentiation of SSC, we established cell culture using a well established protocol^17^. The cultured SSCs, designated as germline stem cells, (GSCs) were derived from isolated Oct4-EGFP+/KIT− germ cells of P5.5 mice and stabilized on mouse embryonic fibroblasts (MEF) as feeder cells (Fig. 1a). In this protocol, the culture medium is supplemented with glial cell-derived neurotrophic factor (GDNF), basic fibroblast growth factor (bFGF/FGF2) and epidermal growth factor (EGF) to sustain long term culture. While GNDF and bFGF are the bona fide self-renewal factors for SSCs, the combination of these growth factors has been demonstrated to be essential for the self-renewal, proliferation and differentiation of SSCs^18,19^. Our culture can be maintained for more than 3 months, suggesting this culture system can support stem cell proliferation. Induction of Receptor Tyrosine Kinase (KIT) expression is a hallmark for spermatogonial differentiation^20^. Interestingly, we found a subset of GSC (~ 20%) in our culture which showed spontaneous differentiation without RA treatment as revealed by KIT expression, albeit at a lower level when compared to the RA treated sample (Fig. 1b,c). This is consistent with the precarious report that spermatogonia cultured with bFGF exhibit more differentiated characteristics such as lower SSC activity and higher KIT expression^21^. Moreover, the EGF in medium could stimulate spermatogonia differentiation^22^. We found that 24 h of RA treatment resulted in a drastic increase in the number of KIT+ cells as revealed by FACS analysis when compared with DMSO control (Fig. 1b,c). We also examined the OCT4 level of RA-treated and control cells, and found that the proportion of GFP negative, GFP low and GFP high cells are similar between control and RA-treated cells (Fig. 1d). Taken together, we demonstrated that RA successfully induced spermatogonia differentiation in our GSC culture.

### Profiling the change in chromatin state after RA treatment in GSC

**Figure 2:**
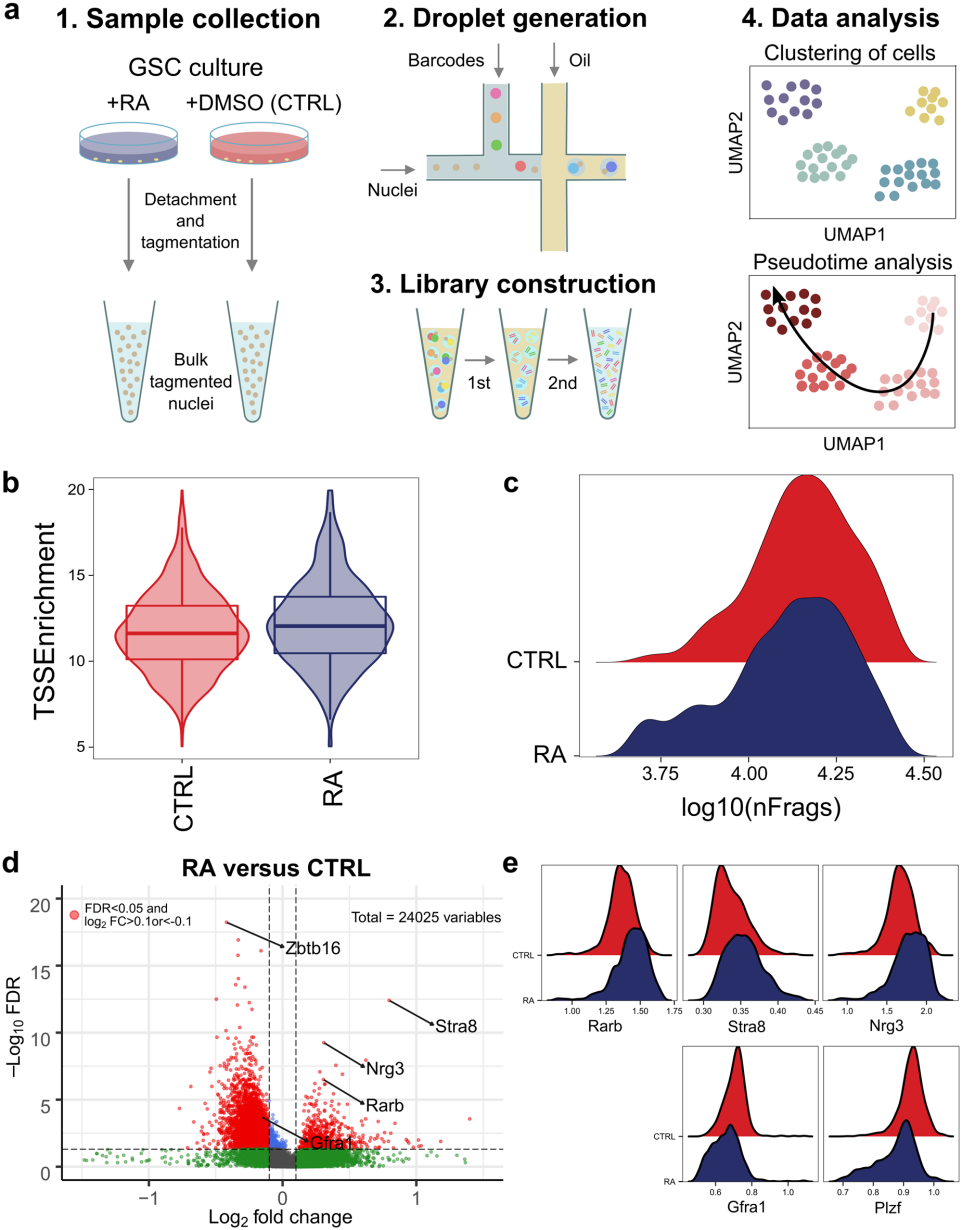
Profiling chromatin accessibility of germline stem cells (GSCs) after retinoic acid (RA) treatment. (**a**) Schematic of experimental design. The workflow of sample collection after RA treatment and scATAC-seq to measure single nuclei accessibility on the BioRad SureCell ATAC-Seq platform. (**b**) Violin plot of TSS enrichment scores. (**c**) Ridge plot of number of unique fragments. (**d**) Pairwise comparison of gene scores between CTRL and RA-treated samples. The volcano plots show the differential gene score against the − log10(P value) of all investigated genes; each dot represents one gene. Red dots indicate the genes with FDR < 0.05 and log2FC > 0.1 or < -0.1. (**e**) Ridge plots of gene activity of SSC self-renewal and differentiation genes in GSCs with (blue) and without (red) RA treatment.

To investigate the cellular heterogeneity and dynamic TF regulation during in vitro spermatogonial differentiation induced by RA, we performed scATAC-seq on the following samples: (1) GSCs treated with vehicle (CTRL); (2) GSCs treated with RA (RA) (Fig. 2a). 48 h after treatment, the culture cells were harvested and the isolated nuclei were subjected to Tn5 tagmentation and single-cell capture with the Bio-Rad SureCell platform. After filtering out low-quality nuclei and doublets, we obtained 2256 and 1888 high-quality single nuclei from CTRL and RA group, respectively (Supplementary Table S1), with a read depth of 14,847 (CTRL) and 10,850 (RA) fragments per nuclei and median transcription start site (TSS) ratio (the percentage of fragments at TSS) of 11.8 (CTRL) and 12.5 (RA) (Fig. 2b,c). The information of individual nuclei is listed in Supplementary Table S2. Marker peak analysis identified that 100 peaks were differentially upregulated and 476 peaks were differentially downregulated after RA treatment (Supplementary Figure S1; Supplementary Table S3). To confirm that RA had effects on the chromatin accessibility of key regulator genes in GSCs, we generated synthetic pseudo-bulk datasets by merging data from cells in control and RA-treated groups to show re-configuration of chromatin (Supplementary Table S4). A gene score is used to predict gene expression based on the accessibility of regulatory elements in the vicinity of the gene, including the gene body and putative distal regulatory elements. Reassuring, we observed that gene score of self-renewal genes (e.g. *Gfra1* and *Plzf*) were decreased after RA treatment while that RA-induced genes (e.g. *Rarb*, *Stra8* and *Nrg3*) increased accessibility (Fig. 2d,e; Supplementary Figure S2)^23^.

### scATAC captures the epigenetic landscape of GSC in vitro

**Figure 3:**
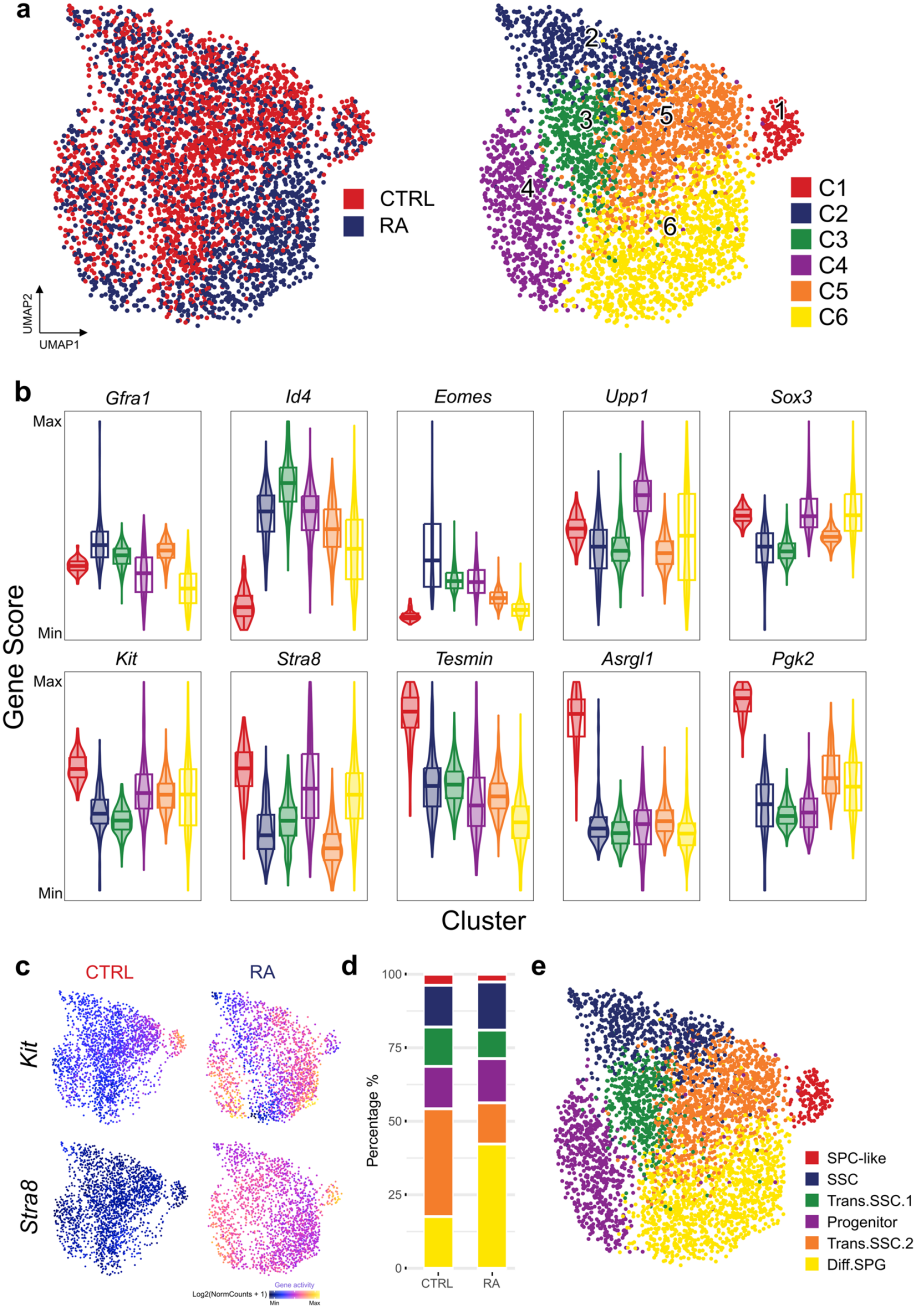
Defining the differentiation status in germline stem cells (GSCs) by gene activity. (**a**) UMAP representation of all cells. Cells are colored by samples (left) and by cell clusters (right). (**b**) Violin plots showing gene activity scores of the selected genes. (**c**) Gene activity scores of *Kit* (top) and *Stra8* (bottom) in CTRL and RA samples shown in UMAP. (**d**) Bar chart showing the distribution of cells in each cluster. (**e**) Annotation of cell states visualized using UMAP from A.

We subjected cells to dimensionality reduction using ArchR and identified 6 major clusters of cells using the graph clustering approach implemented by Seurat (Fig. 3a). We examined the property of the six clusters based on gene scores of known markers (Fig. 3b, Supplementary Figure S3). Cluster 2 (SSC) showed the highest gene scores of SSC marker genes *Gfra1*, *Eomes*^24^ and *Id4*^3^, indicating this cluster showed stem cell properties. SSC marker gene scores in transitional Cluster 3 (Trans.SSC.1) and 5 (Trans.SSC.2) are lower. Cluster 4 (Progenitor) exhibited higher gene scores of progenitor markers (*Sox3* and *Upp1*). Previous studies showed that *Sox3* is not expressed by the steady-state stem cell population (GFRα1-positive) but is rapidly upregulated during initial differentiation commitment steps of stem cells^25^. Cluster 6 (differentiating spermatogonia (Diff.SPG)) showed higher gene scores of differentiation markers (*Kit* and *Stra8*), indicating they are at a more differentiated state. Lastly, Cluster 1 (spermatocyte (SPC)-like) had higher gene scores for spermatocyte-expressed genes like *Tesmin*, *Pgk2*, and *Asrgl1*. It has been reported that RA is sufficient for the in vitro induction of mouse spermatocytes^20^. It is possible that Cluster 1 cells were the more differentiated or spermatocyte-like cells in the culture system. Cluster 1 cells also showed higher gene activity of *Kit*, which may partly contribute to the KIT+ cells as seen in flow cytometry analysis (Figs. 1c, 3c). This subpopulation warranted future investigation of both transcript and/or protein levels of spermatocyte markers in the culture system.

RA caused a significant increase in Diff.SPG cluster by proportion, which suggested that Diff.SPG cluster should be the differentiating cells resulting from RA stimulation. On the contrary, there are more cells in Trans.SSC.1/2 clusters in the control sample. This shift from Trans.SSC.1/2 to Diff.SPG after RA treatment indicates a profound chromatin accessibility change in a subset of GSCs (Fig. 3d). Moreover, although the Progenitor cluster in RA samples showed increased gene activity of *Kit* than CTRL samples, they were still clustered together. This suggested that stem-cell-like subsets (Cluster 3 and 5) have more profound change in global chromatin accessibility after RA treatment than progenitor clusters (Cluster 4) (Fig. 3c).

Taken together, single-cell chromatin accessibility profiling successfully deconvoluted cellular subsets associated with GSC differentiation (Fig. 3e).

### Regulatory regions associated with RA response identified by cisTopic

**Figure 4:**
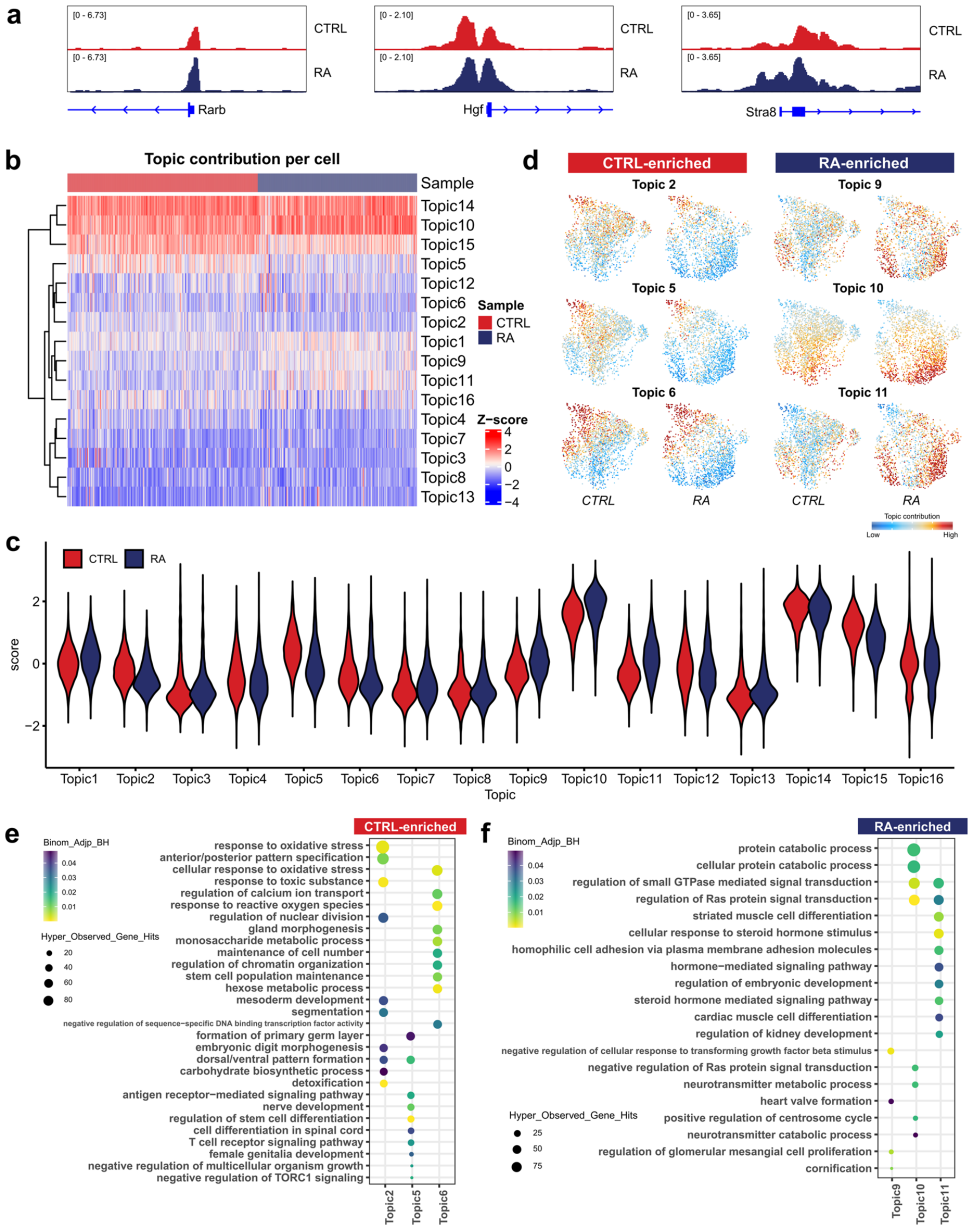
Regulatory topics associated with GSC response to retinoic acid (RA). (**a**) Normalized pseudo-bulk accessibility tracks showing RAR/RXR direct target genes comparing control and RA treatment groups. (**b**) Heatmap showing Topic-cell enrichment revealed by cisTopic analysis. (**c**) Violin plot of the normalized topic score of topics. (**d**) UMAP color-coded by the normalized topic score of selected CTRL-enriched and RA-enriched topics. (**e**) GREAT analysis of regions included in CTRL-enriched topics. (**f**) GREAT analysis of regions included in RA-enriched topics.

Toward uncovering RA-associated chromatin dynamics, we used MACS to call peaks and identify 112,292 peaks in the merged dataset. We examined the distribution of consensus peaks across genomic features in the mouse reference genome. These peaks represent a mixture of cis-regulatory elements including intergenetic regions and promotors, which is consistent with the typical ATAC-seq pattern (Supplementary Figure S4). Comparing the pseudobulk RA sample with CTRL sample revealed that peaks at known RAR/RXR direct target genes *Rarb*, *Hgf* and *Stra8* gain accessibility (Fig. 4a).

We then used cisTopic to investigate RA-induced dynamic changes in chromatin accessibility at single-cell level. cisTopic identified groups of regulatory regions with similar distribution patterns as a “regulatory topic”^26^. The model with 16 topics was selected (Supplementary Figure S5). 16 topics with different enrichment patterns across clusters and samples were identified (Fig. 4b,c). Topics 2, 5 and 6 are more enriched in the CTRL sample, while Topics 9, 10 and 11 are enriched in the RA-treated sample (Fig. 4d). Genomic Regions Enrichment of Annotations Tool (GREAT) analysis revealed that CTRL-enriched topics are associated with stem cell population maintenance, maintenance of cell number and regulation of stem cell differentiation, which is consistent with the function of GSCs (Fig. 4e). On the other hand, RA-enriched topics were associated with cellular response to steroid hormone stimulus and hormone-mediated signaling pathway, suggesting the RA-treated GSCs were more prepared for spermatogenesis (Fig. 4f). For example, Topic 11 includes regions associated with genes in the RA receptor signaling pathway, such as *Cyp26b1*, *Rara*, *Rarb*, *Rarg* and *Rxra*. RA-enriched topics were also related to the protein catabolic process.

### Reconstruction of the stem-to-differentiating transition trajectory

**Figure 5:**
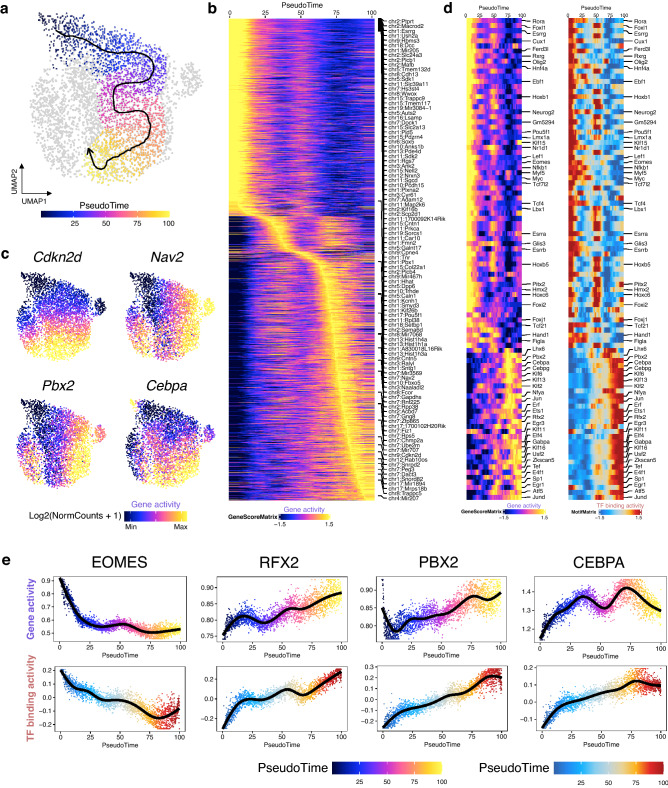
Pseudotime trajectory reveals TF dynamics regulated by retinoic acid (RA). (**a**) scATAC-Seq profiles are ordered by pseudotime, corresponding to the differentiation trajectory induced by RA through Trans.SSC.2. (**b**) Smoothened heatmap depicting genes whose gene score co-varies across pseudotime. (**c**) Gene activity scores of the selected genes shown in UMAP. (**d**) Smoothened heatmap showing dynamic gene score (left) and motif accessibility (right) of indicated TFs along pseudotime for gene-motif pairs of the trajectory. (**e**) Gene activity (top) and motif accessibility (chromVAR TF z-score, bottom) of selected TFs ordered by pseudotime.

We sought to reconstruct the differentiation trajectory using a semi-supervised pseudotime approach. Trans.SSC.2 (Cluster 5) showed a dramatic decrease in cell number in the RA treated group accompanied by the emergence of Diff.SPG (Cluster 6), while the proportion change of other clusters is less profound (Fig. 3d). Therefore, we reasoned that the SSC cluster (Cluster 2) reached the Diff.SPG cluster through Trans.SSC.2. Our data do not exclude the possibility that the differentiation process starts from SSC (Cluster 2) to progenitors (Cluster 4) and finally differentiated cells. However, we decided to focus on the stem-to-differentiating spermatogonia transition (Clusters 2/5 to Cluster 6), which reflects the major cellular dynamic in our RA treatment model. We then generated an ordering of single cells (referred to as ‘pseudotime’) along the trajectory (Fig. 5a). Following that, we identified the gene candidates associated with the transition upon RA induction and revealed a list of genes with varied gene scores across the trajectory (Fig. 5b). For example, *Cdkn2d* is upregulated at the end of the trajectory, and *Cdkn2d* expression has been reported to be significantly induced by RA to promote differentiation in leukemia^27^ (Fig. 5c). *Nav2*, which is RA-inducible in the nervous system to regulate cell migration, is also enriched at the late stage of the trajectory^28^ (Fig. 5c).

To identify critical TFs that are involved in the RA-induced differentiation process, we next correlated the gene score of a TF (infers gene expression/activity) across pseudotime to its corresponding chromVAR TF z-score (infers TF binding activity). In brief, a TF z-score, which is calculated by chromVAR, can infer the activity of a TF based on the abundance of its corresponding motif within open chromatin regions^29^. However, it has a limitation that it could not provide a one-to-one correspondence between binding motifs and particular TF proteins, because TFs of the same family often share a similar motif. We reasoned that a TF with high binding activity should also be more abundant in the cell, which could be accompanied with higher gene expression. Therefore, we identified the TFs with high correlation between gene activity and TF binding activity. This uncovered a list of TFs enriched at different stages of the trajectory (Fig. 5d). TFs from the FOX, MYF and TCF families were identified in the early stages of the trajectory, which is consistent with previous TFs identified in undifferentiated spermatogonia during mouse spermatogenesis^16^. In line with the role of EOMES in SSC maintenance, the gene activity and TF binding activity of EOMES decrease along the trajectory (Fig. 5e). In addition, it has been reported that LEF1 is specifically expressed in undifferentiated spermatogonia and decreased expression of LEF1 led to a down-regulation of PLZF^30^. In contrast, RFX2 showed higher gene activity and TF binding activity at the end of the trajectory. *Rfx2* is crucial for fertility as knockout of *Rfx2* leads to arrest of spermatogenesis in mice in the round spermatid phase of spermiogenesis^31^ (Fig. 5e). Several Krüppel-like factor (KLF) family members also exhibited higher activity at the late stage of pseudotime trajectory. We also identified some candidates that have been reported to respond to RA in other cell types but not in spermatogonia. For instance, *Pbx2* can be induced by RA both transcriptionally and post-translationally in P19 cells^32^ (Fig. 5e). C/EBPα showed higher gene score after RA treatment in leukemia cells, which also induced differentiation^33^ (Fig. 5e). Notably, the gene activities of *Pbx2* and *Cebpa* were upregulated in Diff.SPG, which further implicates its potential role in spermatogonial differentiation induced by RA (Fig. 5c). *Figla* and *Lhx6* are enriched in the trajectory during the intermediate stage. In fact, *Figla* regulates genes related to postnatal spermatogenesis in male germ cells^34^ (Fig. 5d).

## Discussion

Understanding the mechanisms underlying the RA-induced spermatogonial differentiation requires a comprehensive understanding of gene expression programs and the corresponding regulatory mechanisms at the chromatin level. However, to date the studies mainly focused on the changes caused by RA at the transcriptome level. Moreover, the heterogeneous nature of spermatogonia makes it more challenging to decipher this complex process. In this study, we present the first single-cell chromatin landscape capturing spermatogonial differentiation in cultured GSCs.

First, we demonstrated that the chromatin accessibility information is able to identify the cells at stem cell or more differentiating states in culture, and further separate cells that undergo stem-to-differentiating transitions. It is immediately obvious that culture GSCs without RA treatment can already be separated into two subsets. The minor population (Progenitor cluster) harbored a more differentiating signature such as higher gene scores of progenitor markers (*Upp1* and *Sox3*) and showed similar proportions before and after RA treatment.

There are several possible reasons to explain the existence of the Progenitor cluster. First, this population might be traced back to the progenitors or differentiating spermatogonia in vivo isolated along with stem cells when the culture is derived. However, since the culture has been maintained for a long time and the progenitors or differentiating cells could be depleted during this period, this is least likely to happen. Second, it is possible that these two subsets of cells represent two modes of self-renewal. In fact, it has been reported that FGF2 supplementation enabled in vitro SSC expansion without GDNF and the FGF2-supported and GDNF-supported cells showed differences in the self-renewal and differentiation patterns^19^. However, we did not detect an increased gene activity of FGF receptors (*Fgfr1*, *Fgfr2* and *Fgfr3*) in the minor subset. Therefore, we favor the interpretation that they originated from the differentiation of stem cell compartment in culture. The GSCs undergo spontaneous differentiation and the cellular heterogeneity in culture involves a dynamic equilibrium between stem cells and progenitors in the normal condition.

We showed that a subset of stem cells can respond to RA and transform to a more differentiated state, which is signified by a more profound change in global chromatin accessibility when compared to the response of progenitor cells. Using state-of-the-art tools, we identified differentially accessible regions, which may represent cis-regulatory elements of RA-responsive genes.

Regulation of gene expression requires the binding of a TF to the gene promoter and other regulatory elements. In this study, we used different approaches to investigate the TF dynamics during spermatogonial differentiation and revealed known and novel TF regulators. We found several KLFs with increased gene scores and increased TF binding activity in the Diff.SPG by the pseudotime analysis. KLFs are zinc finger-containing TFs that regulate development, proliferation and differentiation and are expressed in a wide range of cell types^35^. Some of the TFs that were identified in our study have been implicated in cell differentiation, such as *Klf2* in osteoblasts and *Klf11* in adipocytes^36–38^. Therefore, it is plausible that these KLFs also drive spermatogonial differentiation.

Our study focused on chromatin accessibility and did not include scRNA-seq analysis of GSCs. Recently, multimodal single cell profiling (“multi-omics”) has greatly improved our ability to detect unique cell types and states. Joint data of scATAC-seq and scRNA-seq from the same cell showed that they are comparable for determining cell identity, suggesting cells coordinate chromatin structure with transcription. Nevertheless, some cell states may not reflect equally in both profiles. Future studies would benefit from joint profiling to provide additional information that further elucidates spermatogonial differentiation. Despite the limitations, our results expand our recognition of the molecular mechanisms and epigenetic regulation underlying the action of RA as the key regulator of spermatogonial differentiation. Our work thus provides a comprehensive resource for understanding the heterogeneity associated with RA response and the TF dynamics related to spermatogonial differentiation in GSCs.

## Methods

### Animals

All animal procedures were performed according to protocols approved by the Animal Experimentation Ethics Committee (AEEC) of the Chinese University of Hong Kong and following the Animals (Control of Experiments) Ordinance (Cap. 340) licensed from the Hong Kong Government Department of Health. All the mice were housed under a cycle of 12-h light/dark and kept in ad libitum feeding and controlled the temperature of 22–24 °C. Oct4-EGFP transgenic mice (B6;CBA-Tg(Pou5f1-EGFP)2Mnn/J, Stock no.: 004654) were acquired from The Jackson Laboratory and maintained in CUHK Laboratory Animal Services Center^39^. All experiments were done in accordance with ARRIVE guidelines and complied with the ethical guidelines of the Animal Experimental Ethics Committee (AEEC) of the Chinese University of Hong Kong.

### SSC isolation and derivation of long-term culture

Spermatogonial stem cells from testes of Oct4-EGFP transgenic mice at P5.5 were purified using the method described previously^17^. Isolated seminiferous tubules were digested with 1 mg/ml type 4 collagenase (Gibco), 1 mg/ml hyaluronidase (Sigma-Aldrich) and 5 µg/ml DNase I (Sigma-Aldrich) at 37 °C for 20 min with occasional shaking. The suspension was passed through a 40-µm strainer cap (BD Falcon) to yield a uniform single cell suspension. After incubation in staining buffer (PBS supplemented with 1% FBS, HEPES, glucose, pyruvate and penicillin–streptomycin) with APC anti mouse CD117 c-KIT antibody (553356, BD Biosciences) at 4 °C for 30 min, Oct4-EGFP+/KIT− cells were collected with a BD FACSAria Fusion Flow Cytometer (BD Biosciences). Primary SSCs were then cultured on the mouse embryonic fibroblast (MEF) layer (150,000 cells per well of a 24 well tissue culture plate). GSC medium (Supplementary Table S5) was used for GSC maintenance. GSCs were passaged by treating with 0.25% trypsin (Invitrogen) for 2 min and adding fresh medium to stop trypsinization. After centrifugation at 1000 rpm for 5 min, the cell pellet was resuspended in medium with a density of 100,000 cells per well of a 24 well tissue culture plate seeded with MEF.

### In vitro retinoic acid treatment and flow cytometry

For retinoic acid (RA) treatment, GSCs were cultured in complete GSC medium containing 0.5 μM RA (R2625, Sigma) for 24 h before flow cytometry analysis. Medium containing DMSO was used as a control.

GSCs were subjected to flow cytometry analysis after trypsin digestion. For analysis, cells were incubated for 30 min at 4 °C in staining buffer with APC anti mouse CD117 c-KIT antibody. Flow cytometry was performed on a BD FACSAria Fusion Flow Cytometer (BD Biosciences). Data were analyzed by FlowJo software (FlowJo, LLC).

### scATAC-seq analysis

#### Sample collection

Cells at passage 5 were used for experiments. For the RA treatment group, 5 wells of cells were seeded on MEF feeder cells and treated with 0.5 μM RA. On the day of the scATAC-seq experiment, the cells were trypsinized and collected. Cells were purified using FACS to remove MEF feeder cells, cell debris and cell aggregate, and pooled together in equal numbers (total 120,000 cells for library construction). For the control group, cells were cultured on the MEF feeder cells and were treated with vehicle (DMSO) for 48 h.

#### Cell lysis and tagmentation

Cell tagmentation was performed according to SureCell ATAC-Seq Library Prep Kit (17004620, Bio-Rad) User Guide (10000106678, Bio-Rad) and the protocol based on Omni-ATAC was followed^40^. In brief, washed and pelleted cells were lysed with the Omni-ATAC lysis buffer containing 0.1% NP-40, 0.1% Tween-20, 0.01% digitonin, 10 mM NaCl, 3 mM MgCl2 and 10 mM Tris–HCl pH 7.4 for 3 min on ice. The lysis buffer was diluted with ATAC-Tween buffer that contains 0.1% Tween-20 as a detergent. Nuclei were counted and examined under microscope to ensure successful isolation. Same number of nuclei were subjected to tagmentation with equal ratio of cells/Tn5 transposase to minimize potential batch effect. Nuclei were resuspended in tagmentation mix, buffered with 1 × PBS supplemented with 0.1% BSA and agitated on a ThermoMixer for 30 min at 37 °C. Tagmented nuclei were kept on ice before encapsulation.

#### scATAC-seq library preparation and sequencing

Tagmented nuclei were loaded onto a ddSEQ Single-Cell Isolator (Bio-Rad). scATAC-seq libraries were prepared using the SureCell ATAC-Seq Library Prep Kit (17004620, Bio-Rad) and SureCell ATAC-Seq Index Kit (12009360, Bio-Rad). Bead barcoding and sample indexing were performed with PCR amplification as follows: 37 °C for 30 min, 85 °C for 10 min, 72 °C for 5 min, 98 °C for 30 s, eight cycles of 98 °C for 10 s, 55 °C for 30 s and 72 °C for 60 s, and a single 72 °C extension for 5 min to finish. Emulsions were broken and products were cleaned up using AMpure XP beads. Barcoded amplicons were further amplified for 8 cycles. PCR products were purified using AMpure XP beads and quantified on an Agilent Bioanalyzer (G2939BA, Agilent) using the High-Sensitivity DNA kit (5067-4626, Agilent). Libraries were sequenced on HiSeq 2000 with 150 bp paired-end reads.

#### Sequencing reads preprocessing

Sequencing data were processed using the Bio-Rad ATAC-Seq Analysis Toolkit (v1.0.1). This toolkit is a streamlined computational pipeline, including tools for FASTQ debarcoding, read trimming, alignment, bead filtration, bead deconvolution, cell filtration and peak calling. The reference index was built upon the mouse genome mm10. For generation of the fragments file, which contain the start and end genomic coordinates of all aligned sequenced fragments, sorted bam files were further process with “bap-frag” module of BAP (https://github.com/caleblareau/bap, v0.6.0). Downstream analysis was performed with ArchR (ArchR_1.0.2)^41^. Fragment files were used to create the Arrow files in the ArchR package.

#### Clustering and gene score/transcription factor activity analysis

We filtered out low-quality nuclei with stringent selection criteria, including read depth per cell (> 2000) and TSS enrichment score (> 4). Potential doubles were further removed based on the ArchR method. Bin regions were cleaned by eliminating bins overlapping with ENCODE Blacklist regions, mitochondrial DNA as well as the top 5% of invariant features (house-keeping gene promoters). ArchR was used to estimate gene expression for genes and TF motif activity from single cell chromatin accessibility data. Gene scores were calculated using the addGeneScoreMatrix function with gene score models implemented in ArchR. addDeviationsMatrix function was used to compute enrichment of TF activity on a per-cell basis across all motif annotations based on chromVAR (chromVAR_0.3). Dimensionality reduction was performed using the addIterativeLSI function and the cell embedding was generated using the addUMAP in ArchR. The clustering was performed using Seurat (Seurat_4.2.0) in ArchR. To identify marker genes based on gene scores, we used the getMarkerFeatures function in ArchR with useMatrix = "GeneScoreMatrix". ArchR performed Wilcoxon testing for comparing the CTRL and RA groups after accounting for the biases (i.e., “TSSEnrichment" score and "log10(nFrags)". These P values are then adjusted for multiple hypothesis using the FDR method. The volcano plot was generated by EnhancedVolcano package (https://github.com/kevinblighe/EnhancedVolcano).

#### cisTopic analysis

Topic modeling was performed using 10–35 (1 by 1), 40 and 500 iterations with cisTopic (cisTopic_0.3.0)^42^. The model was selected based on the highest log-likelihood. The cell-topic UMAP representation was obtained by using UMAP on the normalized topic-cell matrix (by Probability). Cell clustering result (6 clusters) from ArchR was used for visualization. Heatmap was then generated based on the cell-cisTopic distributions to identify topics associated with each cluster. To select a representative set of regions of the topic, Region-topic distributions were binarized with a probability threshold of 0.99 to identify the top contributing regions in each topic. For identifying enriched GO terms per topic, the binarized topics (i.e. sets of top regions per topic) were analyzed over GREAT (rGREAT_1.22.0) or clusterProfiler (clusterProfiler_3.18.1)^43,44^.

#### Trajectory analysis

Trajectory analysis was performed in ArchR. addTrajectory function in ArchR was used to construct trajectory on UMAP embedding. To perform integrative analyses for identification of positive TF regulators by integration of gene scores with motif accessibility across pseudo-time, we used the correlateTrajectories function which takes two SummarizedExperiment objects retrieved from the getTrajectories function.

### Github repository

The analytical codes used in this study are available on the GitHub repository (https://github.com/liaojinyue/SSC_culture_RA_scATAC).

### Statistical analysis

Assessment of statistical significance was performed using two-tailed unpaired t-tests, one-way ANOVA with Tukey multiple comparisons tests or Chi-squared tests. Statistical analysis was performed using GraphPad Prism v8. Associated P values are indicated as follows: *P < 0.05; **P < 0.01; ***P < 0.001; ****P < 0.0001; not significant (ns) P > 0.05.

## Supplementary Information

Supplementary Information 1.

Supplementary Information 2.

Supplementary Information 3.

Supplementary Information 4.

## Data Availability

The datasets generated and/or analyzed during the current study are available in the NCBI Gene Expression Omnibus (GEO) repository, under accession number GSE167531 (https://www.ncbi.nlm.nih.gov/geo/).
