# Supplementary material for: scATAC-Seq reveals heterogeneity associated with spermatogonial differentiation in cultured male germline stem cells
Source: Sci Rep. 2022 Dec 12;12:21482. doi: 10.1038/s41598-022-25729-7 (PMC9744833; doi:10.1038/s41598-022-25729-7)
Supplement: Supplementary file 1 — Supplementary Information 1. [file 41598_2022_25729_MOESM1_ESM.docx]

***Supplementary Material***

## **Supplementary Tables**

| **Sample** | **Number of nuclei passed filter** | **Median of unique fragments per cell** | **Median of TSS enrichment score** |
| --- | --- | --- | --- |
| RA | 1888 | 10850 | 12.5135 |
| CTRL | 2256 | 14847 | 11.843 |

#

# **Supplementary Table S1.** Quality control metrics of scATAC-Seq libraries.

**Supplementary Table S2.** Metrics of individual nuclei passing quality control in scATAC-Seq libraries (in separate spreadsheet).

**Supplementary Table S3.** Peaks with differential accessibility (FDR < 0.1) between CTRL and RA-treated samples using pseudobulk scATAC-Seq datasets (in separate spreadsheet).

**Supplementary Table S4.** Pairwise comparison of gene scores between CTRL and RA-treated samples using pseudobulk scATAC-Seq datasets (in separate spreadsheet).

|  | **10ml media** | **Work concentration** | **Storage** | **Company** |
| --- | --- | --- | --- | --- |
| StemPro | 8.55ml | - | 4C | Life Technologies |
| Human GDNF | 20ul | 40ng/ml | -80C | R&D Systems |
| Human bFGF | 10ul | 10ng/ml | -80C | BD |
| Mouse EGF | 5ul | 20ng/ml | -80C | Life Technologies |
| StemPro-Nutrient Supplement | 0.26ml | - | -80C | Life Technologies |
| BSA | 200ul | 0.20% | -20C | MP Biochemicals |
| GlutaMax | 100ul | 1x | -20C | Life Technologies |
| ES-FBS | 100ul | 1% | -20C | Life Technologies |
| Peni/Strep | 100ul | 1× | -20C | Life Technologies |
| NEAA | 100ul | 1× | -20C | Life Technologies |
| Sodium pyruvate | 100ul | 1× | -20C | Life Technologies |
| Transferrin | 100ul | 100μg/ml | -20C | Sigma |
| VITAMIN | 100ul | 1× | -20C | Sigma |
| D(+)-Glucose | 100ul | 1mg/ml | -20C | Sigma |
| D-biotin | 100ul | 10μg/ml | -20C | Sigma |
| Insulin | 25ul | 25μg/ml | -20C | Sigma |
| NaOH | 18ul | - | R.T. | Sigma |
| Progesterone | 15ul | 60ng/ml | -20C | Life Technologies |
| DL-Lactic acid | 10ul | 1μl/ml | -20C | Sigma |
| Ascorbic acid | 10ul | 100μM | -20C | Sigma |
| β-Estradiol | 10ul | 30ng/ml | -20C | Sigma |
| β-ME | 9.1ul | 50μM | 4C | Sigma |
| Putrescine | 6ul | 60μM | -20C | Sigma |
| Sodium selenite | 1ul | 30nM | -20C | Sigma |

#

# **Supplementary Table S5.** GSC culture medium composition.

#

## **Supplementary Figures**

## **
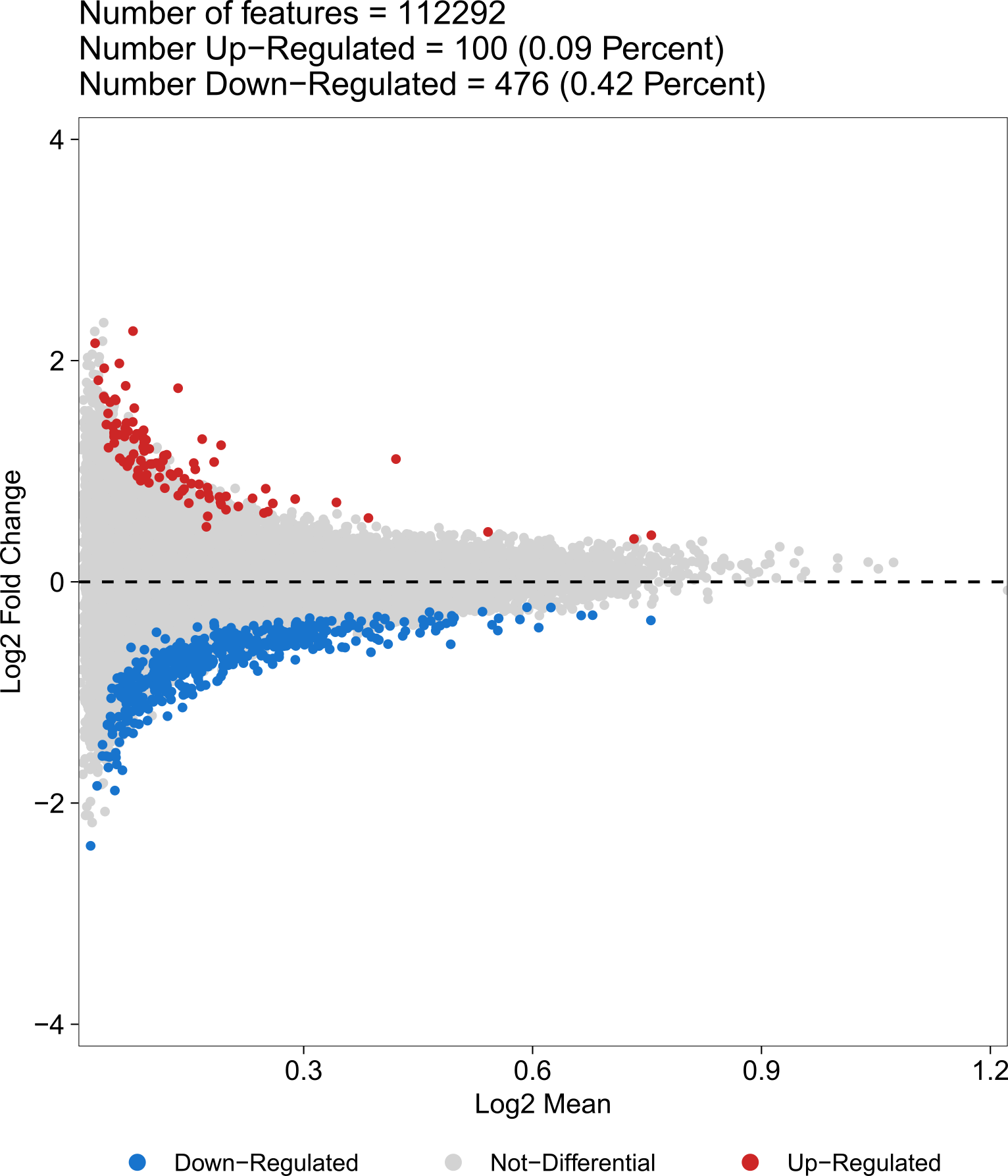
**

## **Supplementary Figure S1. scATAC-seq peak-level analysis, related to Figure 2.** MA plot showing the peaks with differential accessibility (FDR < 0.1). Red dots indicate the peaks upregulated in the RA sample. Blue dots indicate the peaks downregulated in the CTRL sample.

##

## **
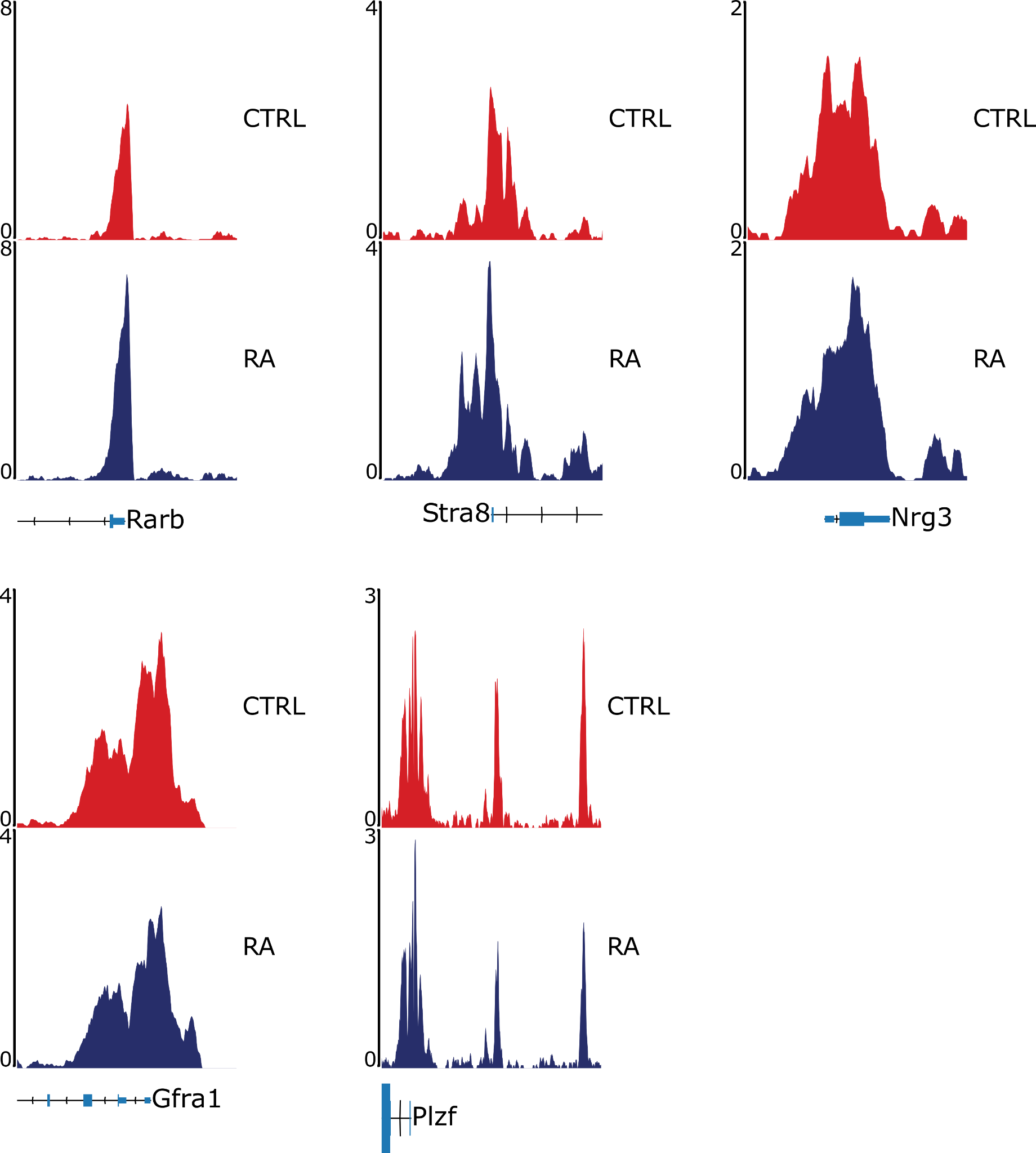
**

## **Supplementary Figure S2. Chromatin accessibility of self-renewal genes and RA-induced genes, related to Figure 2.** Normalised pseudo-bulk accessibility tracks showing the selected genes comparing control and RA treatment groups.

##

## **
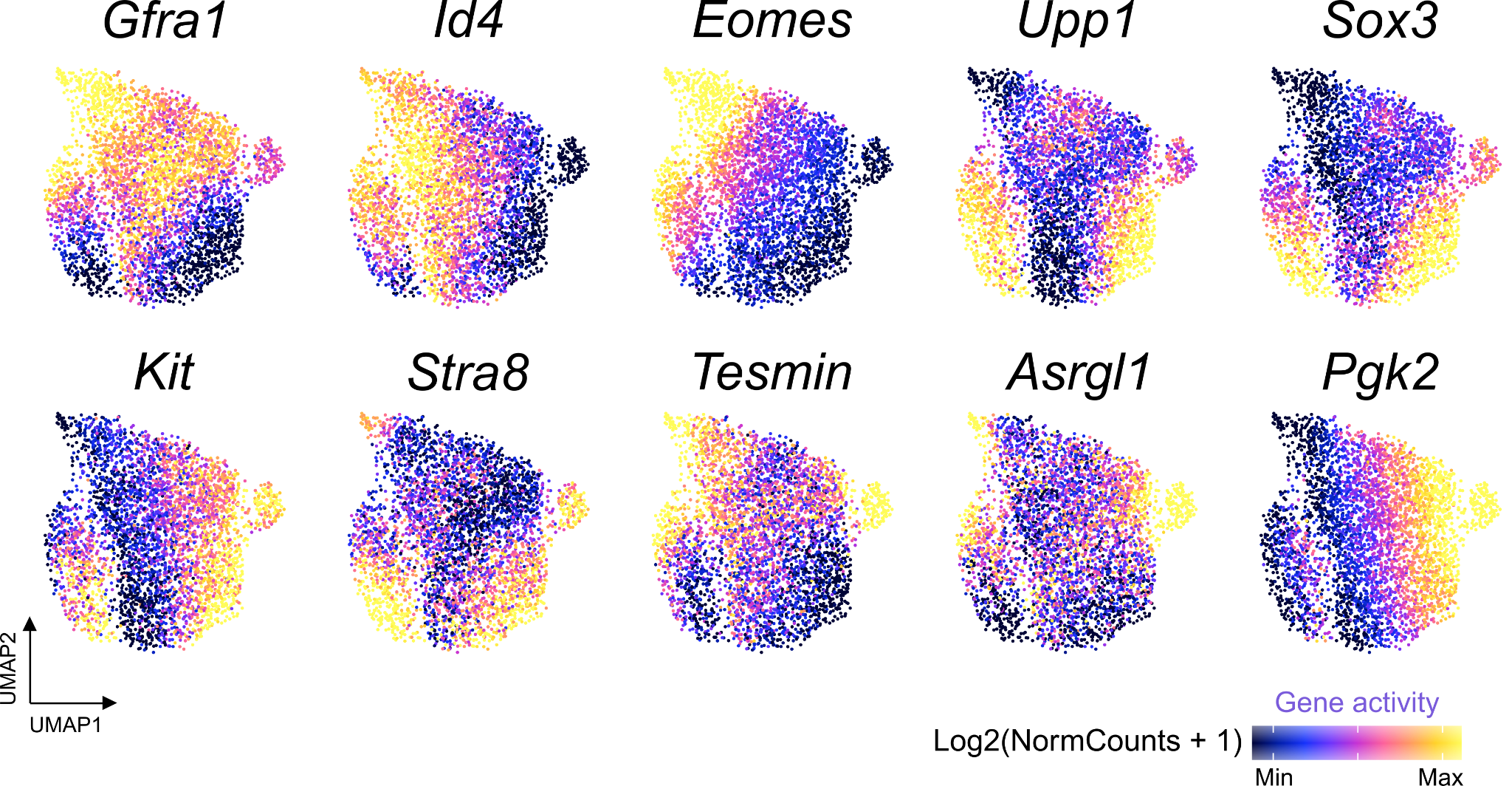
**

**Supplementary Figure S3. Gene activity scores of spermatogonia-related genes, related to Figure 3.**Gene scores of the selected genes shown in UMAP.

#
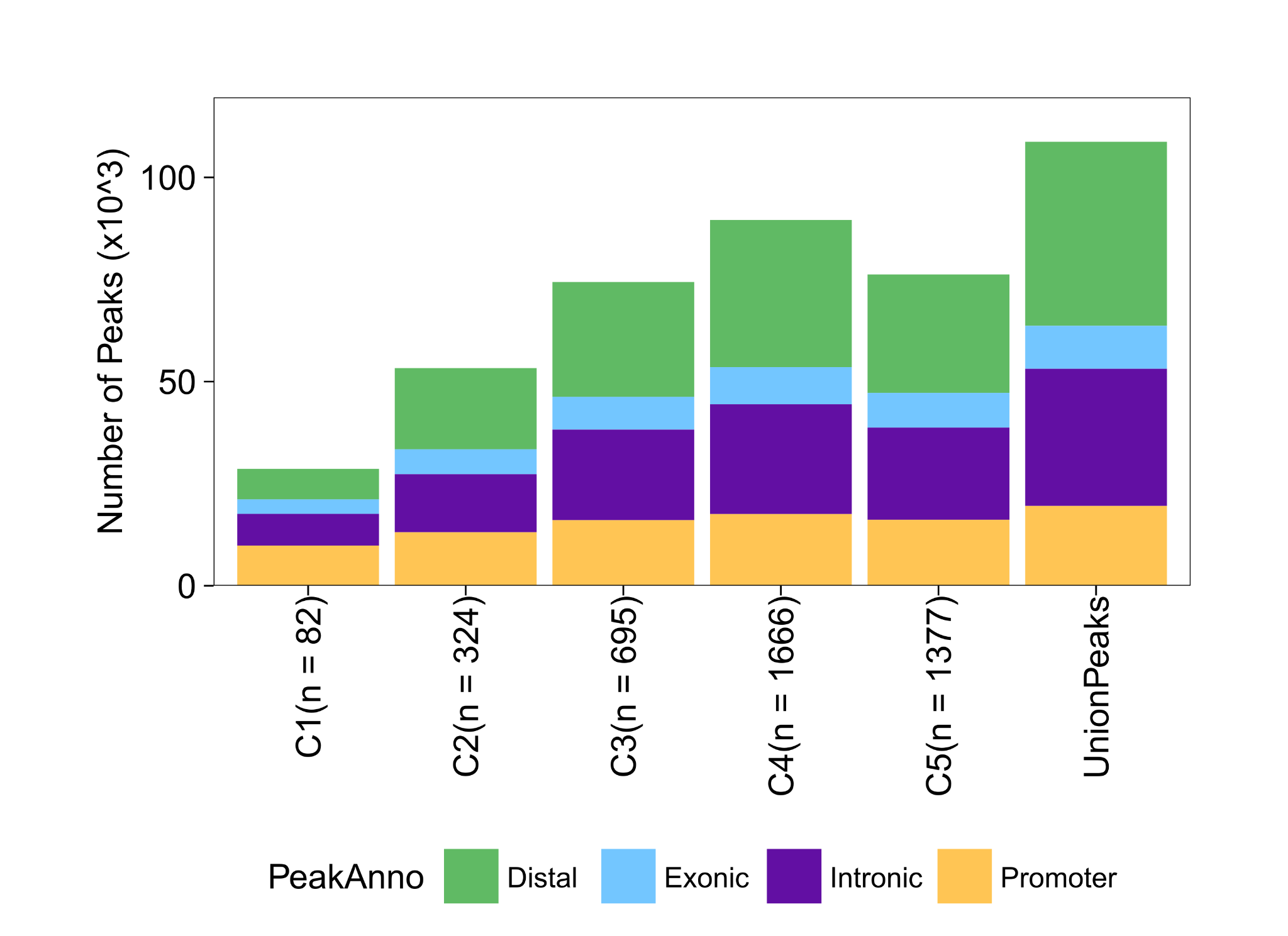


# **Supplementary Figure S4. The distribution of peaks across genomic features, related to Figure 4.** Bar chart showing the distribution of 112,292 peaks across genomic features.

#
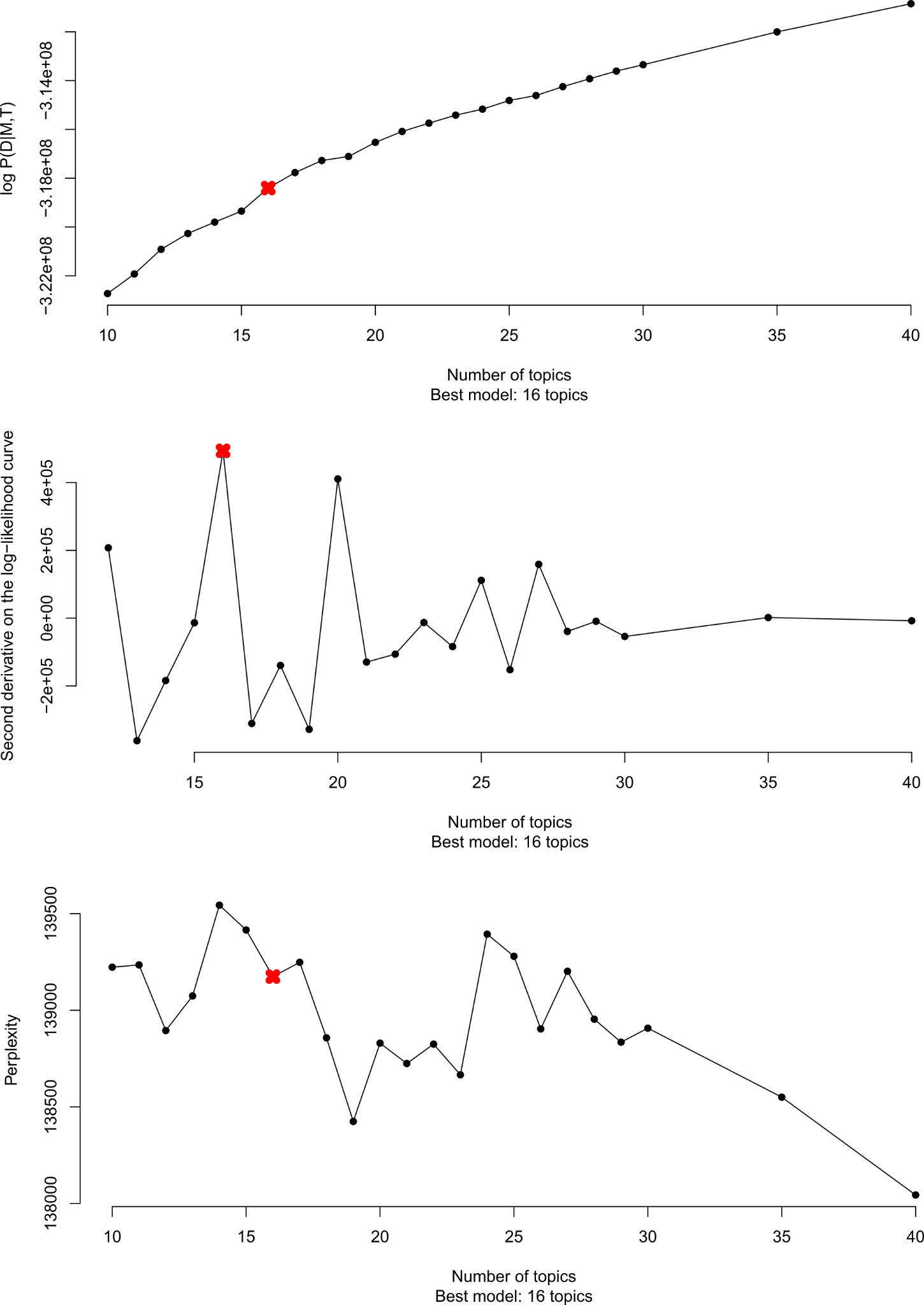


# **Supplementary Figure S5. The cis-Topic likelihood plot for model selection, related to Figure 4.** Plots indicating the model with 16 topics is selected. Automatic selection of the best number of topics was performed using the “selectModel” function with default settings.
